# Pulmonary strongyloidiasis associated CD3+ large granular lymphocytosis

**DOI:** 10.4103/1817-1737.78432

**Published:** 2011

**Authors:** Muhammad A Rishi, Saleha Z Chaudhry

**Affiliations:** *Rosalind Franklin University of Medicine and Science, North Chicago, IL, USA*; 1 *Mercy Hospital Medical Center, Chicago, IL, USA*

**Keywords:** Bronchoalveolar lavage, large granular lymphocytosis, strongyloidiasis

## Abstract

We report a case of pulmonary strongyloidiasis in a patient with large granular lymphocytosis. He was on short-term high dose immunosuppressant therapy. A 77-year-old white male presented to the emergency room with fever and shortness of breath for 10 days. The patient had been diagnosed about 3 months prior to this presentation with “large granular lymphocytosis” (LGL) after a workup for pancytopenia. Methotrexate and prednisone had been started 1 month ago for the treatment of LGL. Five days prior to the current presentation, he had been started on moxifloxacin as an outpatient but got progressively worse and came to an emergency room. Bronchial washings (bronchoalveolar lavage) demonstrated numerous filariform larvae of *Strongyloides stercoralis*. The patient was treated with ivermectin and improved. Pulmonary strongyloidiasis should be considered in the differential if X-ray findings show a interstitial or alveolar pattern and if the patient has visited the endemic areas, even in the remote past.

Strongyloidiasis is a parasitic disease that is usually caused by one of the two species (*Strongyloides stercoralis* and *Strongyloides fuelleborni*) of strongyloides, an intestinal nematode.[1] It is widely distributed in the tropical and subtropical regions.[2] The organism is endemic to former Czechoslovakia[2] but not to Northeastern United States. In immunocompetent individuals, the infection may cause mild gastrointestinal symptoms.[3] Untreated, the infection persists for many decades. Strongyloides hyper infection syndrome develops in patients who subsequently develop immunodeficiencies.[2,3] Pulmonary strongyloidiasis is a common manifestation of strongyloides hyperinfection syndrome.[4,5]

## Case Report

This 77-year-old Czech man, previously a farmer, recently diagnosed with CD3+ LGL by bone marrow biopsy and flow cytometry, presented to the ER with a temperature of 104 °F and mild shortness of breath.

History of present illnessshowed that current symptoms had been present for 1 week. He was started on moxifloxacin as an outpatient without improvement of symptoms. The patient had been living in the Northeastern United States for the past 30 years and had never since traveled to areas endemic to strongyloides. The patient had worsening anemia over 2 months before current presentation secondary to LGL which prompted immunosuppressive therapy (prednisone 20 mg PO three times a day and methotrexate 20 mg once a week) about 4 weeks before the current presentation.

His medical history was significant for CD3+ LGL and hypertension. The patient had a history of low-grade pancytopenia and had been followed by a hematologist as an outpatient. A bone marrow biopsy 3 months before this admission showed increased lymphocyte aggregates. Flow cytometry detected CD3+ T-cell population. T-cell clonality analysis was negative, and so secondary disease was suspected. Common etiologies of secondary LGL [Epstein Bar virus (EBV), Cytomegalovirus (CMV), Hepatitis B virus (HBV), Hepatitis C virus (HCV), Human immunodeficiency virus (HIV), Idiopathic thrombocytopenic purpura (ITP) and Rheumatoid arthritis (RA)] were ruled out and the cause of this nonclonal expansion of large granular lymphocytes remained elusive. Subsequently, the patient was started on immunosuppressive therapy. The patient had no history of dermatological or gastrointestinal manifestations of strongyloides infection.

**Figure 1:**
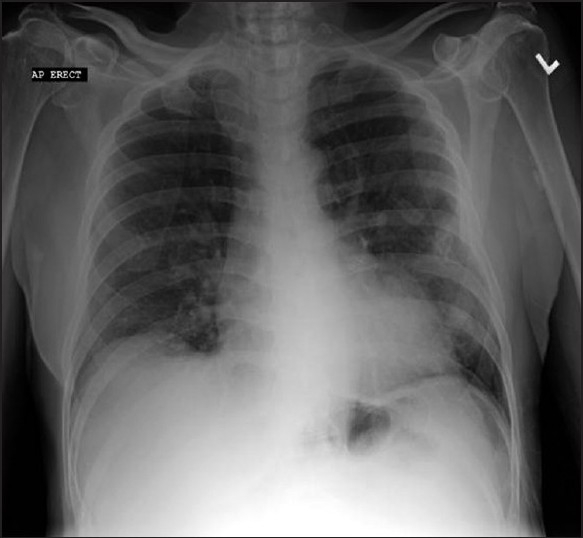
Chest X-ray on admission

Physical exam at the time of presentation was significant for a temperature of 101.4°F, a heart rate of 114/min, respiratory rate of 24/min and oxygen saturation of 94% on 2 L/min oxygen. The patient had a blood pressure of 110/60. Bilateral coarse crackles were heard at the bases of the lungs. Rest of the exam was within normal limits. Laboratory tests on admission are shown in Table 1. Imagingat admission included a CXR [Figure 1] showing increased interstitial markings and low lung volumes. Differential diagnosis included methotrexate-induced pneumonitis, atypical pneumonia (AP), and pneumocystis pneumonitis.

**Table 1:** Admission labs

WBC	5.5	K	4.2
HB	7.9	Cl	97
HCT	32	HCO_3_	25
PLT	223	BUN	35
MCV	85.4	Cr	1.1
NEU%	67.5	GLU	124
LYM%	24.0	Ca	7.8
EOS%	3.5	PHOS	2.3
BAS%	0.1	ALB	2.3
BILI	0.4	AST	38
ALK PHOS	76	ALT	75

**Figure 2:**
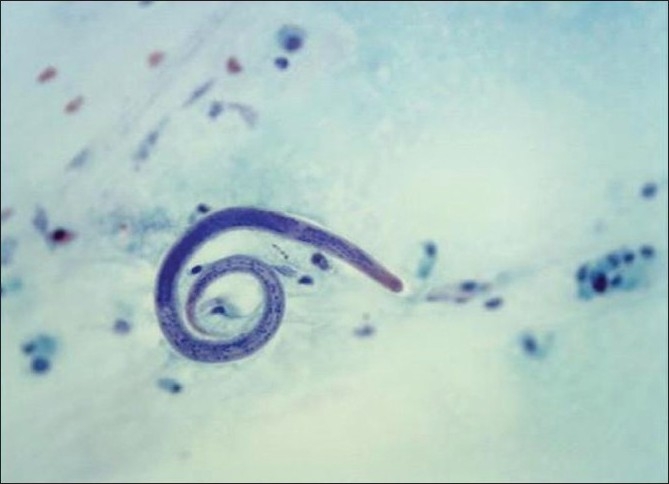
Bronchoalveolar lavage photomicrograph showing *Strongyloides stercoralis*

Hospital course was eventful. The patient was started on ceftriaxone, azithromycin, and bactrim. Prednisone was changed to hydrocortisone 30 mg four times daily after taking blood, urine, and sputum cultures. Methotrexate was stopped. The cultures remained negative over the next 48 h. However, the patient failed to improve. He continued to have persistent hypoxia and spiking fever. On day 3 post-admission, bronchoscopy with bronchoalveolar lavage (BAL) was done. It showed 5-20 S. *stercoralis* per high power field and atypical pneumocytes [Figure 2]. CD4/CD8 ratio was 0.8 (normal range, 0.8-2.1). HTLV 1/2 serology was negative. Tests for HIV also came back negative. Stool was negative for ova and parasites. Strongyloides antibodies were not obtained. The patient was started on Ivermectin 12 mg orally daily for 3 days and improved. The patient was discharged after 11 days of hospital course on a tapering dose of prednisone. Follow-up bronchoscopy was negative for strongyloides after 6 months. Repeat bone marrow biopsy done after 18 months which failed to show CD3+ LGL.

## Discussion

This case is unusual in terms of possible association of pulmonary strongyloidiasis with CD3+ LGL. To our knowledge, this has not been described previously.

Large granular lymphocytes are morphologically recognizable subset of lymphocytes and have slightly basophilic cytoplasm with azurophilic granules. They represent 10–15% of the peripheral mononuclear cells. There are two phenotypes (CD– and CD3+ cells). CD3+ phenotype is the predominant form.[6] A syndrome characterized by proliferation of large granular lymphocytes, i.e. LGL associated with neutropenia was initially reported in 1977[7] and several studies have been published since.[8,9] LGL can be primary or secondary and may be associated to a wide range of hematological and nonhematological disorders. Primary LGL shows clonal expansion and secondary LGL does not.[8] Since the CD3– cells do not rearrange the T-cell receptor gene, and cytogenetic abnormalities are unusual in CD3-LGL expansions, clonality is hard to prove.[10] This is not the case with CD3+ cells. Secondary LGL is considered reactive. EBV, CMV, HBV, HCV, HIV, ITP, connective tissue disorders such as rheumatoid arthritis, skin disorders such as pyoderma gangrenosum and hemophagocytosis syndromes have been described as potential causes of reactive LGL.[8,11,12] One previous case report[10] has described strongyloides as a potential cause of CD3-LGL.

Untreated, strongyloides may persist for many decades as a low-grade chronic infection. Immune suppression (due to corticosteroid therapy as in our patient, malnutrition, leukemia or lymphoma, and HTLV 1 infection) in patients with low-grade strongyloides infection may result in a hyper-infection syndrome.[3,4] Pulmonary strongyloidiasis is one of the manifestations of the hyper-infection syndrome.[5] Acute and subacute forms have been described. Prognosis is usually poor.[13]

However, the diagnosis of pulmonary strongyloidiasis is often difficult and delayed since symptoms and laboratory findings are nonspecific. Chest imaging reveals nonsegmental patchy areas of consolidation diffuse a reticulonodular pattern and diffuse patchy opacities.[14]

It is interesting to note that T/B cell clonality analysis in this patient was negative for any clonal rearrangements. This finding is suggestive of a reactive disorder. Common causes of reactive LGL were ruled out in our patient. Helminthes induce immunity by helper T-cell induced responses.[15] It may be suggested that chronic low-grade stimulation by a low-grade strongyloides infection over many years resulted in a reactive CD3+ large granular lymphocytosis (LGL) seen in this patient.

Resolution of the CD3+ LGL with treatment of strongyloides infection strongly suggests that the chronic strongyloides infection can result in a nonclonal expansion of LGL which resolves if the infection can be treated successfully.
